# Facilitators and Challenges to Adoption of a Digital Health Tool for Opioid Use Disorder Treatment in Primary Care: Mixed Methods Study

**DOI:** 10.2196/69953

**Published:** 2025-07-10

**Authors:** Omar Nieto, Allison D Rosen, Mariah M Kalmin, Li Li, Steven J Shoptaw, Steven P Jenkins, Zahra Zarei Ardestani, Bengisu Tulu

**Affiliations:** 1 Department of Family Medicine, University of California, Los Angeles, 10880 Wilshire Boulevard, Suite 1800, Los Angeles, CA, 90024, United States, 1 3107940229; 2 UCLA Center for HIV Identification, Prevention, and Treatment Services (CHIPTS), University of California, Los Angeles, Los Angeles, CA, United States; 3 Department of Behavioral and Policy Sciences, RAND Corporation, Santa Monica, CA, United States; 4 Department of Psychiatry and Biobehavioral Sciences, University of California, Los Angeles, Los Angeles, CA, United States; 5 Q2i LLC, Boston, MA, United States; 6 Department of Data Science, Worcester Polytechnic Institute, Worcester, MA, United States; 7 Department of Social Science and Policy Studies, Worcester Polytechnic Institute, Worcester, MA, United States; 8 The WPI Business School, Worcester Polytechnic Institute, Worcester, MA, United States

**Keywords:** opioid use disorder treatment, digital health tools, primary care clinics, implementation barriers, system integration

## Abstract

**Background:**

The United States is facing an opioid overdose epidemic resulting in an unprecedented number of preventable deaths. The use of medications including buprenorphine and methadone has proven effective for opioid use disorder (OUD), but many patients struggle to stay in treatment. Novel solutions, such as digital health tools, offer one option to help improve clinic management and improve treatment engagement.

**Objective:**

Using a mixed methods approach, we investigated facilitators and barriers to the use of a third-party digital health platform called Opioid Addiction Recovery Support (OARS) to aid OUD treatment engagement and adherence in a primary care setting.

**Methods:**

Patient and provider use of OARS was observed for 10 months and summarized using descriptive statistics. Differences in use were assessed using Wilcoxon signed rank tests. Additionally, key informant interviews were conducted with providers who prescribe medication for opioid use disorder (MOUD) and their case managers to understand the facilitators and barriers to implementation. Qualitative data were analyzed using a coding reliability thematic analysis approach.

**Results:**

Among 205 patients invited to use OARS, the median age was 37 (IQR 31-44) years, 130 (63.4%) identified as men, and 193 (94.1%) identified as non-Hispanic White. Of these 205 patients, 158 (77.1%) used the app at least 1 time. The median number of days the 158 patients viewed test results was 1 (IQR 1‐3), progress was 1 (IQR 0‐2), and educational content was 0 (IQR 0‐1). The 55 patients whose providers had manually entered their results into OARS when the electronic health record (EHR) integration failed viewed test results (*P*=.002), progress (*P*<.001), and educational content (*P*<.001) more days than the 103 patients who could not view their results in OARS. Providers and the lead case manager reported that OARS increased patient-provider communication, allowed patients to better track their overall MOUD treatment, and enhanced providers’ ability to identify patients at risk for relapse. They also acknowledged that the lack of integration between OARS with the EHR resulted in administrative burdens, which impacted provider use of the system.

**Conclusions:**

Findings from this study highlight the challenges of successfully implementing OARS with patients who receive MOUD in primary care settings. Our results show a lack of OARS uptake among providers, case managers, and patients, despite positive assessments made by participants. We also show several barriers that impacted provider use, including the lack of integration between OARS and EHR. Future research is needed (1) to determine whether digital health tools like OARS are efficacious in improving OUD outcomes and, if proved efficacious, (2) to identify ways to routinize the use of digital health tools in MOUD treatment, primarily by solving technical and organizational challenges associated with EHR integration and patient engagement.

## Introduction

The opioid overdose epidemic in the United States continues to claim many lives. In 2022, there were 6.1 million people with opioid use disorder (OUD) [1], and opioid-involved deaths rose from 49,860 in 2019 to 81,806 in 2022 [2]. Medication for opioid use disorder (MOUD) with buprenorphine or methadone is the most effective treatment to reduce opioid use and is associated with reduced risk of overdose and opioid-related acute care use [3,4]. Facilitating retention in MOUD is critical. Studies have shown that the risk of death is 8.1 times lower for patients who are in MOUD compared to those who do not receive MOUD [5,6]. However, many patients discontinue MOUD treatment [7], with more than 50% of these patients relapsing [8].

Integrating MOUD into primary care settings may be an optimal strategy to expand access to treatment. Korthuis et al [9] suggest that offering OUD treatment in primary care settings may be particularly advantageous for those individuals already engaged in care or who struggle with accessing opioid treatment programs (eg, methadone clinics). Unfortunately, providers encounter multiple barriers to delivering MOUD in primary care settings, which include challenges to administrative logistics (ie, orienting new patients and compressed appointment schedules), provider discomfort discussing MOUD with patients, and lack of provider buy-in regarding the expansion of MOUD in the clinic [10]. As such, innovative strategies are needed to address these barriers and enhance the delivery of MOUD in general medical settings.

Digital health solutions (eg, mobile health apps) offer a complementary service delivery modality for MOUD provided in primary care settings to help improve clinical management and long-term patient engagement [11]. Such solutions can also help improve access to treatment and reduce psychosocial barriers [11]. In addition, multiple studies evaluating the acceptability of digital technology interventions have shown a high level of engagement or willingness to use these technologies among patients receiving MOUD services and their providers in diverse health care, academic, and outpatient treatment settings [12-14]. These studies describe many of the prospective benefits of digital health solutions for the management of MOUD care, including improved communication between patients and providers, patients’ ability to track treatment progress, and the prevention of overdose-related deaths [12-14]. However, as noted by Miller-Rosales et al [15], additional work is needed to understand facilitators to the use of these platforms to maximize the potential benefits. As such, the goal of this study was to assess the facilitators and barriers to using a third-party technology system as a strategy to support MOUD services specifically within a primary care setting.

## Methods

### Ethical Considerations

The institutional review board of the University of California, Los Angeles approved all study procedures (IRB #20‐001516-AM-00004). All participants provided written informed consent prior to the interview and received a US $50 electronic gift card for completing each interview. All data were deidentified prior to data analysis.

### Study Design

We partnered with a primary care clinic that serves as an Opioid Use Disorder Center of Excellence in the Eastern United States (hereafter referred to as the “Center”) and followed the implementation of the Opioid Addiction Recovery Support (OARS) platform in this clinic as part of a federally funded project. This project was a pragmatic observational study in a real-world setting, and the research team was not directly involved in implementation.

### OARS Software

OARS was developed by Q2i (a digital health company based in Boston, Massachusetts) as a digital health platform designed to improve the clinical management of MOUD treatment by allowing providers to view records relevant to their patients’ recovery journey, communicate with their patients through secure messaging, and track patients’ progress via a provider dashboard; the app measures progress by tracking appointment attendance and engagement during visits. In addition, patients were given access to the OARS mobile app that allowed them to track their appointment attendance, appointment participation (ie, engagement), and urine drug screening results. Patients were also able to document their personal feelings or stressors through an electronic daily log that providers could view. Finally, the mobile app allowed patients to view their upcoming MOUD appointments, view prepopulated educational content (ie, YouTube videos from the Substance Abuse and Mental Health Service Administration that provide information on triggers, cravings, and recovery), and directly communicate with members of their care team (eg, providers and case managers) through secure messaging. The enhanced connection and support afforded by OARS have the potential to increase adherence to treatment plans, lead to better retention in care, and reduce negative outcomes such as relapse and overdose. While OARS can operate as a standalone platform, this study was designed with the intention to integrate OARS into the Center’s electronic health record (EHR) prior to its implementation with patients. Unfortunately, EHR integration was ultimately unsuccessful, resulting in a multitude of challenges for the Center (described in the Results section).

### Implementation Setting

During implementation, the Center had 28 Drug Addiction Treatment Act-2000 waivered providers (eg, medical doctors, physician assistants, and nurse practitioners), offered MOUD treatment to 200+ patients with OUD, and had an established EHR. Based on information provided by Center staff during project check-ins, the majority of patients at this clinic were maintenance patients rather than patients who had recently initiated treatment. As part of onboarding, Center providers attended a 1-hour orientation via Zoom (Zoom Video Communications) with Q2i staff to familiarize themselves with the dashboard and patient app. The research team also worked with the Center during this time to ensure that all regulatory processes were complete before implementation began (eg, establishing a Reliance Agreement). In addition, Q2i staff and the research team worked with the Center to integrate OARS with their EHR.

After onboarding, providers at the Center were tasked with using OARS for a period of 10 months (from May 1, 2021, through February 28, 2022) with their patients and to participate in monthly check-in meetings with the research team to discuss project updates (eg, barriers or challenges to patient engagement with OARS). The Q2i team was available to address any technical concerns that arose during the project. Of note, OARS was promoted as a tool that could support patient recovery, but it was not mandatory for patients to sign up for an OARS account.

### Data Collection

Patient and provider use of OARS was observed for the entire 10-month implementation period. Data collected through the OARS platform included the date and time that each feature of the OARS app was used (ie, viewing urine drug screening results, viewing MOUD appointment attendance, sending secure messages, and creating daily logs). Additional patient-level data were collected through EHR extraction, which included patient demographic information, appointment attendance, and urine drug screen results. All data were deidentified by Q2i staff prior to data analysis.

In addition to the quantitative data captured through OARS, the research team also invited MOUD providers and their case managers to participate in a series of key informant interviews 1 month prior to OARS implementation, 2 to 3 months after initial implementation, and during the postimplementation period (ie, at least 10 months from the initial start date) to further assess the use of OARS overtime. Participation in the interviews was voluntary for all staff. Participants were recruited during optional monthly check-in meetings with the research team and were then separately connected with the project coordinator to schedule an interview date over Zoom. To be eligible, providers had to prescribe MOUD treatment to their patients and have a Drug Addiction Treatment Act-2000 waiver (required during the time of the study). Conversely, case managers had to work closely with an eligible provider at the Center to participate in the interviews.

As part of the interview, providers and case managers were asked to describe (1) what they liked and disliked about the OARS platform, (2) their thoughts about the usability of OARS (ie, whether OARS is easy or difficult to use), and (3) the barriers or challenges they encountered to getting started using OARS. All interviews were transcribed verbatim by Rev and reviewed for accuracy by an evaluation team partner (ON).

### Exclusion of Patients on MOUD in the Qualitative Evaluation

Due to our institutional review board protocol, the research team was prohibited from having any contact with patients. As such, these individuals were not directly recruited into the study. Instead, providers were asked to introduce OARS to their patients on MOUD and invite them to use the platform to help manage their care. However, patients were allowed to opt out of using OARS without penalty. Only Q2i staff were allowed to have direct contact with patients to address technical issues, while the research team maintained a strictly observational role during OARS implementation.

### Data Analysis and Measures

The primary quantitative outcome in this analysis was the number of days patients interacted with OARS, including the number of days patients interacted with each specific feature. This was measured by tabulating the number of days that patients interacted with the platform and by plotting a timeline for each individual patient who logged into OARS more than 1 time.

In addition, the number of days of interaction with each OARS feature was compared for patients whose providers had manually entered their results into OARS and patients whose providers had not entered their results and thus did not have any results to view (ie, test results, appointment attendance, and treatment progress). We used a Wilcoxon signed rank test to make this comparison. Despite the efforts of the research team, integrating OARS with the EHR system used in the Center was not successful. Lack of integration between the 2 systems resulted in providers having to manually enter test results into OARS for the patients to view the results through their app. However, providers did not always enter test results into OARS. This meant that some patients were not able to view their results in OARS, and we hypothesized that this could partially explain the lower-than-expected patient engagement with OARS throughout the study period.

The aforementioned procedure was also used to compare feature interaction for patients who were and were not consistently engaged in MOUD treatment during the study period, hypothesizing that patients who were consistently engaged in MOUD would be more likely to interact with OARS. Because patients were expected to engage in treatment at least once per month to receive a new 28-day prescription for MOUD, treatment engagement was defined as not having a greater than 35-day gap (1-week grace period) in appointment attendance or urine drug testing during the study period, as measured by EHR data provided by the Center.

The number of days patients created daily logs was not included in these comparisons due to insufficient sample size. A Bonferroni correction was applied to all *P* values by multiplying each *P* value by 4, the number of tests conducted, to correct for multiple comparisons; a *P* value less than .05 was considered statistically significant after correction.

Timelines of OARS use were also described for MOUD providers and case managers. All analyses were conducted in R (version 4.2.1; R Foundation for Statistical Computing). All qualitative data were analyzed using a coding reliability thematic analysis approach [16]. We first created a codebook consisting of both deductive codes from the interview guide and inductive codes from a line-by-line review of the transcripts. Next, the research team coded 2 transcripts to refine code descriptions, add or delete codes, resolve discrepancies in the interpretation of codes, and identify exemplar quotes associated with each code. This process continued until the research team reached a consensus on the code application. In total, 2 authors (ie, ON and ZZA) and 1 additional support staff then independently coded an additional transcript in Dedoose (a qualitative data management software) to run a test of intercoder reliability, and an average Cohen κ score was computed (κ=0.85). All remaining transcripts were uploaded into Dedoose and coded using the finalized coding scheme. As part of the analysis, we sought to assess provider buy-in, the usability of the system, and the benefits of specific OARS features to providers and patients. Finally, we sought to identify barriers to implementation that arose during the project.

## Results

### Overview

A total of 205 patients were invited to use OARS. The median age was 37 (IQR 31‐44) years, 130 (63.4%) identified as men, and 193 (94.1%) identified as non-Hispanic White. Of these 205 patients, 158 (77.1%) signed up for an account and used the app at least 1 time. Patients who used OARS tended to be younger than patients who did not use OARS (Table 1).

**Table 1.** Patient demographic characteristics.

	Invited to use OARS^a^ app (N=205)	Signed up for OARS account	
		Yes (n=158)	No (n=47)
Age (years), median (IQR)^b^	37 (31‐44)	36 (31‐44)	39 (31‐44)
Man, n (%)	130 (63.4)	101 (63.9)	29 (62)
Non-Hispanic White, n (%)	193 (94.1)	147 (93)	46 (98)

a OARS: Opioid Addiction Recovery Support.

b *P*<.001.

More specifically, 76 patients interacted with OARS for 1 day, and 82 interacted with OARS for more than 1 day; the maximum number of days a patient interacted with OARS was 26 days (Table 2).

**Table 2.** Number of days patients interacted with OARS^a^ (N=205), May 2021 to February 2022.

Total days of OARS use	Values, n (%)
0	47 (22.9)
1	76 (37.1)
2	20 (9.8)
3	11 (5.4)
4	17 (8.3)
5	10 (4.9)
>5	24 (11.7)

a OARS: Opioid Addiction Recovery Support.

**Figure 1.**
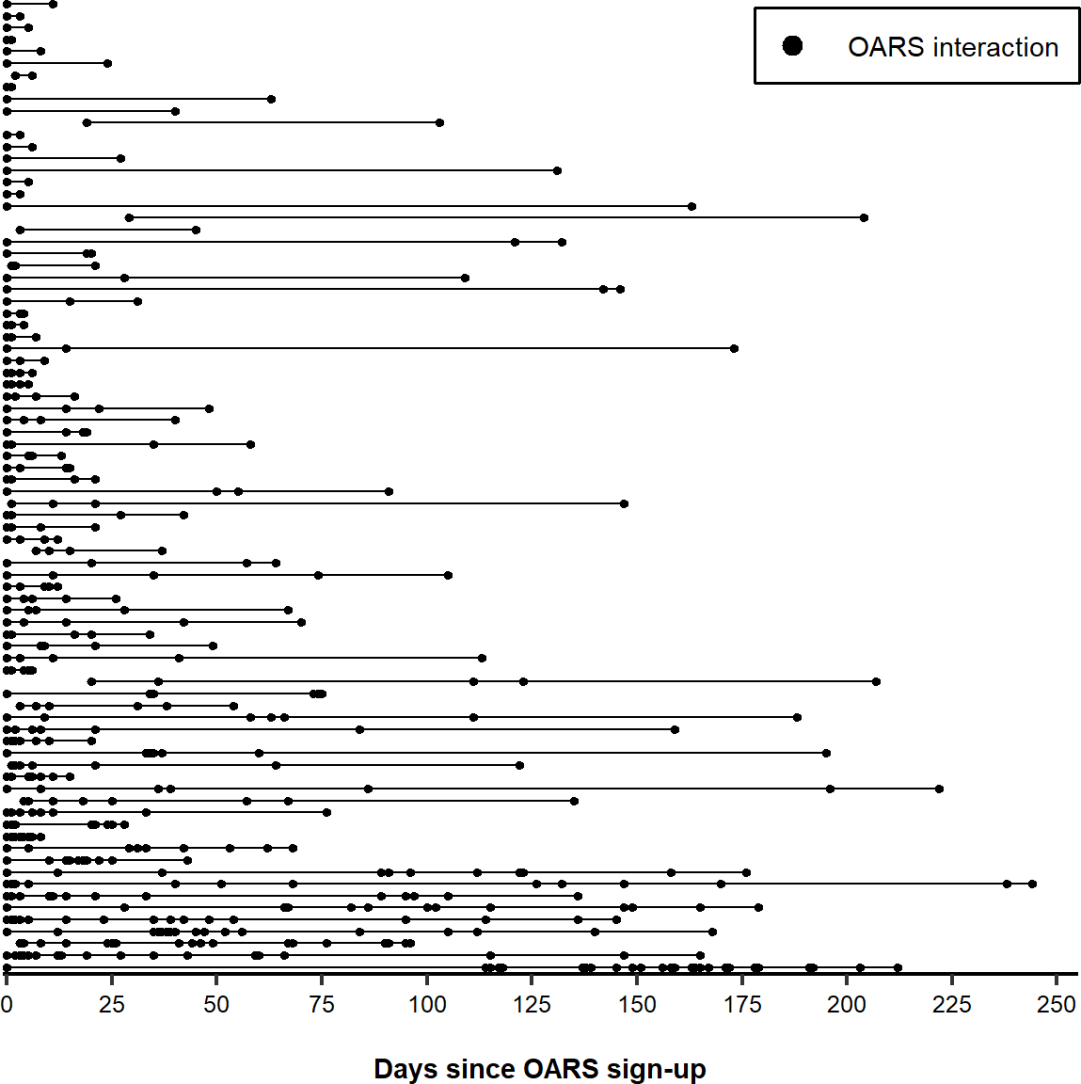
Daily OARS interactions by patients who interacted with OARS at least 2 days over the course of the study. Each horizontal line represents 1 patient, and each point represents the use of OARS (n=82), May 2021 to February 2022. OARS: Opioid Addiction Recovery Support.

Among the 82 patients who interacted with OARS for more than 1 day, use patterns varied over the course of the study (Figure 1). For example, among patients who interacted with OARS for 4 days or less, some used it for a few days after signing up, while others’ use was spread over the course of the study.

**Figure 2.**
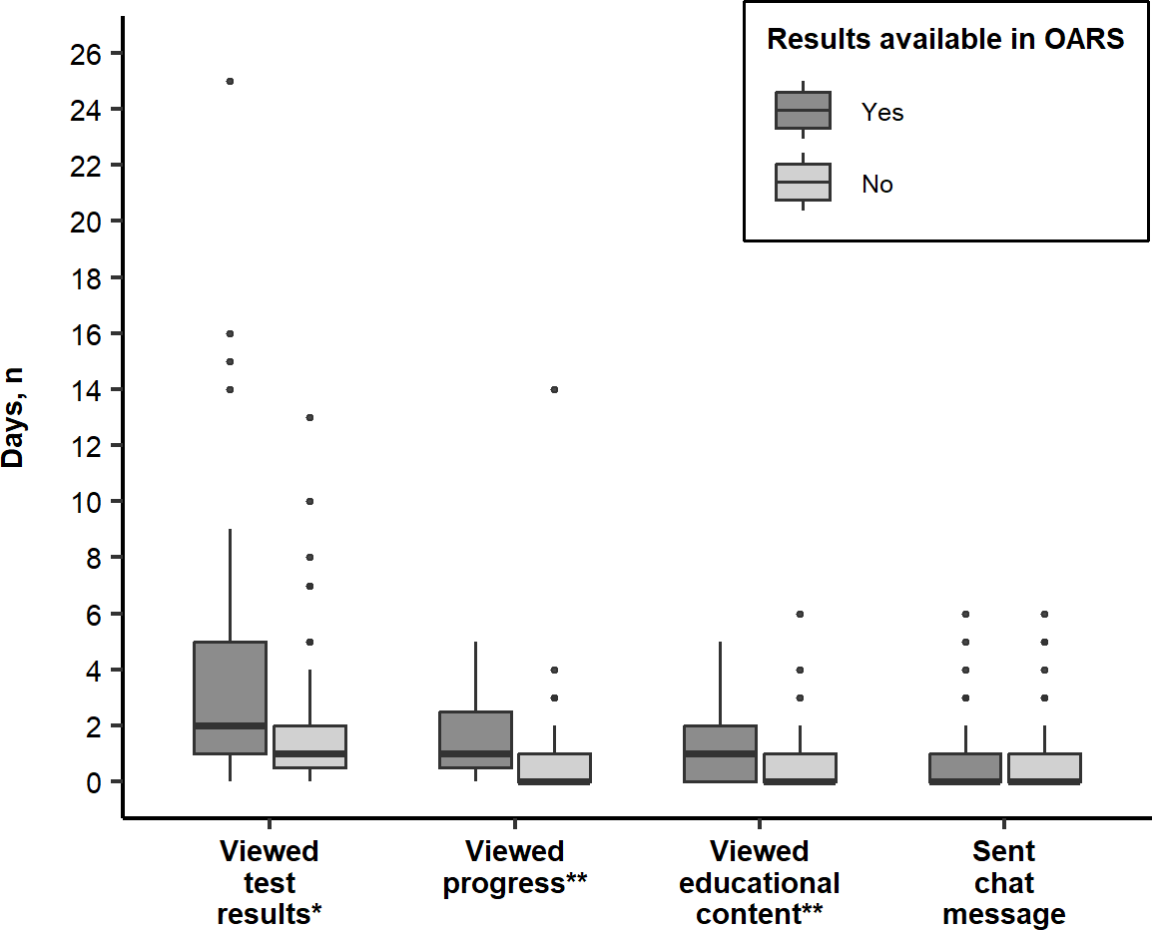
Comparison of the number of days of interaction with each OARS feature for patients who had manually entered their results into OARS and patients whose providers had not entered their results and thus did not have any results to view (n=158). OARS: Opioid Addiction Recovery Support. *P=.002,**P<.001.

**Figure 3.**
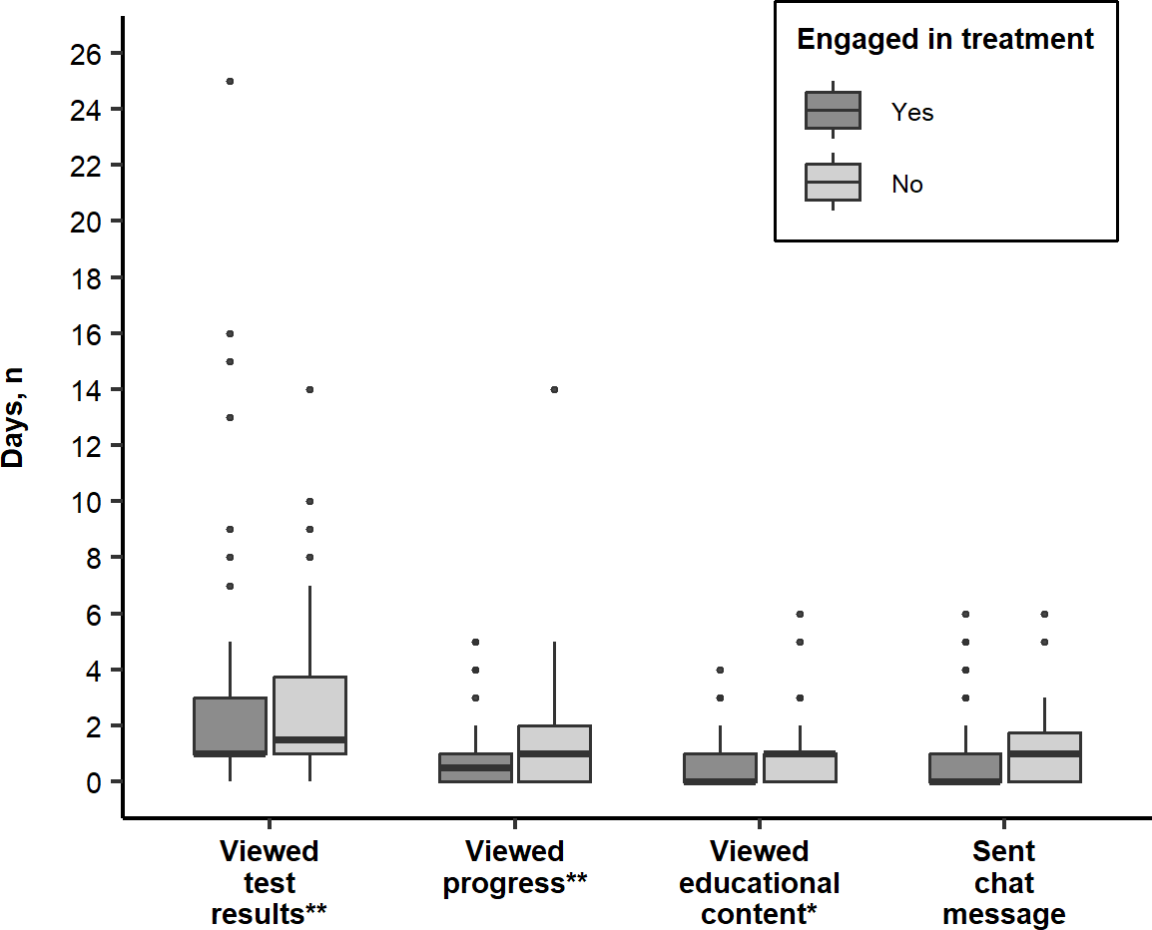
Comparison of the number of days of interaction with each Opioid Addiction Recovery Support feature by consistent treatment engagement status (n=112). *P=.003,**P<.001.

Over the course of the study period, the median number of days the 158 patients viewed test results was 1 (IQR 1‐3), progress was 1 (IQR 0‐2), educational content was 0 (IQR 0‐1), sent a chat message was 0 (IQR 0‐1), and created a daily log was 0 (IQR 0‐0). Of note, the 55 patients whose providers had entered their results into OARS viewed test results (*P*=.002), progress (*P*<.001), and educational content (*P*<.001) more days than the 103 patients who could not view their results in OARS (Figure 2). Additionally, among 112 patients who also had treatment engagement data available in their EHR, the 34 patients who were not consistently engaged in MOUD treatment viewed test results (*P*<.001), progress (*P*<.001), and educational content (*P*=.003) on more days than the 78 patients who were consistently engaged in MOUD treatment (Figure 3).

The messaging and daily log features had the lowest use; 69 (44%) patients used the messaging feature at least 1 time. Of these 69 patients, 28 (40%) sent more than 1 message, and the maximum number of messages sent was 6. Finally, among 12 (8%) patients who created daily logs, 3 (12%) made more than 1 entry (maximum 4 entries).

**Figure 4.**
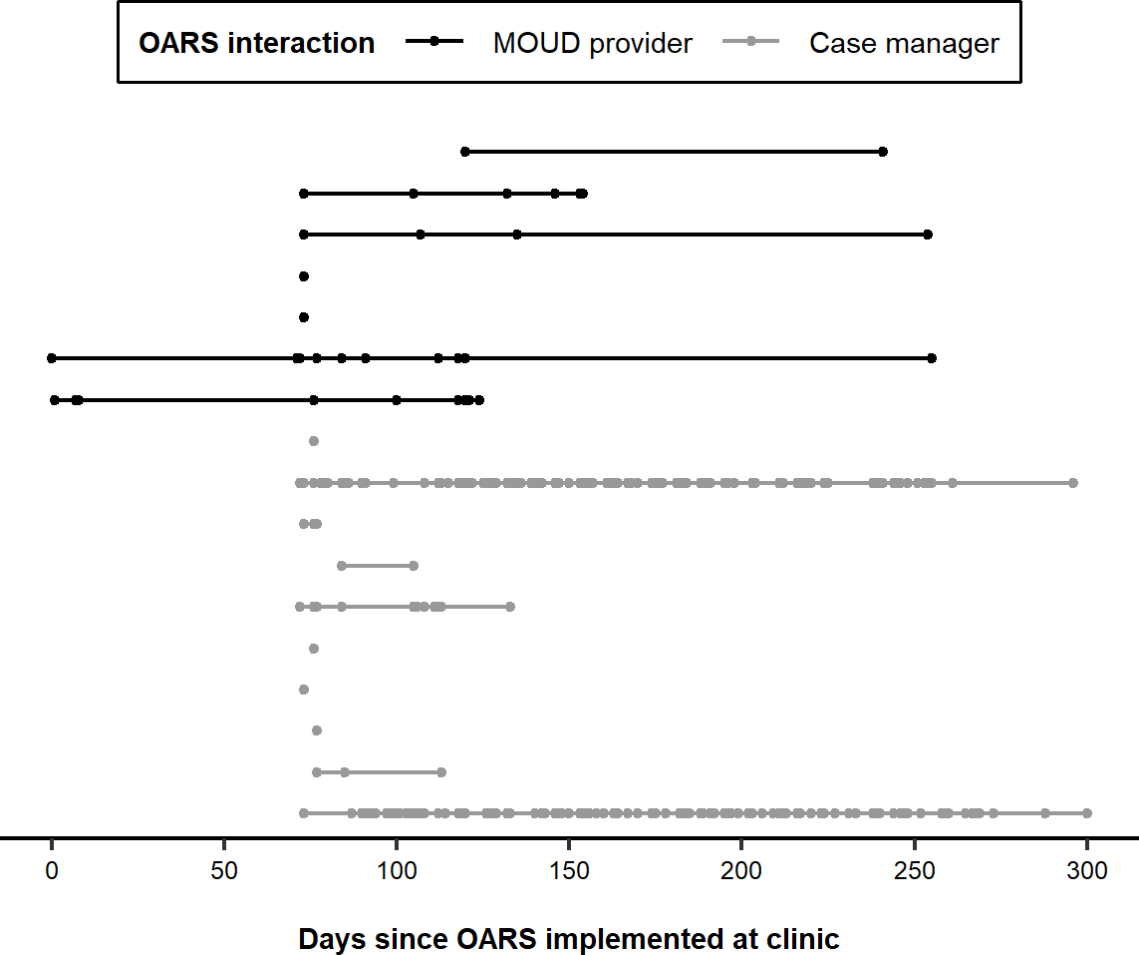
Daily OARS interactions by MOUD providers and case managers (n=17), May 2021 to February 2022. Each horizontal line represents 1 MOUD provider or case manager, and each point represents interaction OARS. MOUD: medication for opioid use disorder; OARS: Opioid Addiction Recovery Support.

Among the 28 staff at the Center invited to use OARS, 7 MOUD providers and 10 of their case managers interacted with the system at least once during the study period. In total, 2 MOUD providers began interacting with OARS at the beginning of implementation, but most did not begin regular use until patient recruitment increased during the third month of the study. Of the 7 MOUD providers, 5 used the system between 2 and 10 days, and 2 used OARS on only 1 day. Among case managers, 8 used OARS up to 11 days, and 2 used OARS over 90 days (Figure 4).

### Facilitators to OARS Use

A total of 8 staff members who indicated interest in using OARS were invited to participate in interviews (6 MOUD providers and 2 case managers), but only 3 agreed to participate. Among those 3, 2 were MOUD providers and 1 was the lead case manager. Interviews took place 1 month prior to implementation (n=3), soon after initial implementation (n=3), and during the postimplementation period (n=2). One of the MOUD providers opted out of the final postimplementation interview.

In general, providers and the lead case manager who participated in the interviews believed that OARS improved overall satisfaction at the Center by increasing communication among providers, case managers, and patients, as illustrated in the following quotes:

I think both parties see some improvement in that, in satisfaction. Case managers are more positive about seeing it as a tool that they can use. And patients are seeing it in a more positive light in regards to ease of communication to the case managers.

[Lead case manager, second interview]

I think both parties see some improvement in that, in satisfaction. Case managers are more positive about seeing it as a tool that they can use. And patients are seeing it in a more positive light in regards to ease of communication to the case managers.

[Lead case manager, second interview]

OARS was also described as being easy or simple to use, straightforward, self-explanatory, and convenient. When discussing specific features, secure messaging appeared to be the most useful feature for providers because it provided their patients with a convenient way to get in touch with members of their care team and freed up time that providers would otherwise spend fielding calls. In addition, secure messaging allowed providers to both manage the high volume of patients in their caseload and respond to patient needs in a timely manner:

And then the communication by using the chat box, that was really efficient for my patients. Because with the amount of patients I have and the other responsibilities, it just made it easy for them to contact me rather than trying to even get me by phone. Because I would get a text message that I had a message on OARS ... . So, it was a lot easier to get back in touch with a patient quickly.

[Lead case manager, third interview]

Of note, providers did not gain much utility from viewing urine drug screening results or appointments in OARS because they already had access to this information in the EHR. Nonetheless, staff believed that patients benefited most from these features. As one provider notes, urine drug screening results were “important for their [patients’] day-to-day life where they have a consistent record of how are they doing” (Provider A, second interview). In addition, providers believed that OARS was especially important for patients who are forgetful and have trouble remembering upcoming appointments:

For me, the least important for me, like I said before, because I’m the one setting up the appointments, I don’t have to know. I already know when they are [scheduled to come back]. But for the patients, that’s important. To them, that’s important because they have a tendency to forget when their appointments are. You can tell them today and then tomorrow [they may call back to ask about the appointment].

[Lead case manager, second interview]

Finally, the daily logs were valued by providers, despite their limited use among patients, because it gave patients an opportunity to vent frustrations about their treatment (eg, long wait times or delayed prescriptions) or communicate issues they might not otherwise feel comfortable discussing in person. More importantly, these daily logs allowed providers to identify patients who were at risk for potential relapse and allowed for early intervention:

I felt the daily log was very useful because that is something that can be powerful, that we can use over time, because it could really predict bad outcomes. Like if you feel like someone is not doing well mentally for several days in a row, that patient is really high risk for relapse. So, I feel that daily log could help us in predictive analysis and predict relapses ...

[Provider A, second interview]

### Barriers to OARS Use

Despite the potential benefits of OARS, several barriers emerged that impacted the use of the system. These barriers included challenges with the specific technology, lack of buy-in due to manual data entry, and access-related difficulties.

The primary technology-specific barrier was related to the OARS patient onboarding process. When patients first signed up with the system, providers were required to manually assign them to members of their care team in OARS. However, the system did not remind staff to assign newly registered patients to a care team member. Patients who were unassigned were thus unable to directly communicate with members of their care team, resulting in a potential gap in treatment.

Lack of buy-in from some staff and patients emerged as another key challenge to the use of OARS, which was influenced by several factors. First and foremost, the lack of full technology integration between OARS and the existing EHR system forced providers and their case managers to manually upload patient appointments and urine drug screening information into OARS, which diminished enthusiasm:

Yeah, there was a lot of manual data entry, like the next appointment date has to be manually entered. The urine test has to be manually entered, the results. There was no interface [between OARS and the data in EHR]. So, I think that manual data entry, if that could be eliminated, it could be better utilized.

[Provider A, third interview]

This manual entry requirement resulted in late or missed appointments or urine drug screening entries, which was thought to impact the use of the app by patients.

Recurring check-in meetings with the technology partner, Q2i, provided greater context for understanding these technological challenges. Namely, the Center’s EHR system was not cloud-based and lacked open application programming interfaces required for full integration with OARS. In the absence of full EHR integration—the ultimate purpose of the study—the Center relied on uploading relevant patient data (ie, appointments and test results) through a secure file transfer connection in predefined intervals. In general, this data transfer took 1 to 7 days to populate in OARS depending on the resources available to manually pull data from the EHR and upload it into OARS. Because using OARS required duplicate manual entry, providers believed that it was largely an “administrative” burden to the Center and were less likely to use the system.

Providers also believed that some features offered in OARS were redundant to the existing patient portal, which already allowed patients to view their test results and appointments and securely message providers. In addition, providers believed that the availability of similar services across multiple platforms made it confusing for patients to know when to use OARS versus the patient portal and understand the utility that OARS offered compared to what already existed at the Center. Providers also believed that some patients were hesitant to use OARS due to potential privacy-related concerns. As the lead case manager indicates, patients “were just so paranoid that they thought this is how the government’s tracking them” (second interview).

Finally, providers noted that patients encountered multiple challenges to accessing the technology. For example, their patients often struggled with or were unable to use OARS because they would lose their phone or lacked access to a phone, a reliable connection to the internet, or the funds to pay for minutes on their mobile devices:

Some of my patients struggle with always having cell phone or smartphone accessibility. So, for them, it was a bit of a challenge. Whether they’re out of minutes or lost their phone or whatever it may be. So, that posed a different challenge for them.

[Provider B, second interview]

## Discussion

### Principal Findings

Our results highlight many of the challenges to successfully implementing OARS with patients who receive MOUD in primary care settings. As was often the case, a discrepancy emerged between our qualitative (ie, staff members’ positive assessments of OARS) and quantitative findings (ie, lack of uptake among providers, case managers, and patients). For instance, the 2 core benefits of OARS reported by staff were that it increased patient-provider communication and allowed patients to track their overall MOUD treatment progress, which was in alignment with other studies that evaluated similar digital health solutions [12,17,18]. However, the messaging feature was underused by patients, with most patients only sending a message once to providers. This suggests that providers and case managers should have taken a more active role in initiating conversations or check-ins with patients rather than relying on passive engagement from the latter.

Providers also valued the additional information that OARS offered about patients through the daily logs, which allowed them to identify patients who were at risk for relapse. However, a discrepancy emerged yet again between providers’ positive evaluation of the daily logs and their use by patients, which was quite low in this study. While it may be important to collect self-reported information on risk factors (ie, cravings) and other relevant life stressors (eg, divorce and work termination), it is clear from our data that additional or alternative features were needed to keep participants engaged. For example, prior research suggests that patients on MOUD might benefit from being able to directly track cravings through a mobile app like they would with urine drug screening results and appointments [19,20]. In addition, technology interventions could also benefit from actively engaging patients through recurring wellness prompts and motivational coaching, as noted in the existing literature [19,21]. Such additions may have greatly enhanced the utility of OARS for both patients and their providers.

In addition, several notable barriers emerged that impacted the use of OARS and warrant further discussion. Our study revealed that provider buy-in was largely impacted by a lack of integration between OARS and the existing EHR. Literature reports that EHR integration is a common challenge facing third-party solutions and should be considered, as health systems evaluate multiple products into their organization’s IT infrastructure [22]. In our case, enthusiasm for OARS and frequency of interaction with the platform was diminished by the administrative overhead experienced during implementation due to the lack of full EHR integration.

The experience of implementing OARS at the Center provided valuable insight that primary care clinics should consider when seeking to enhance the delivery and management of MOUD services through the use of a third-party technology system. First and foremost, these settings will need to ensure that they have the necessary technical capacity or infrastructure (ie, open application programming interfaces) in place to integrate with systems like OARS before implementation begins [23]. Second, both the health care system and third-party technology partner need to invest the necessary resources to ensure seamless technology integration [24]. Otherwise, the resulting administrative burdens might outweigh the potential benefits of using the platform. It will also be important to develop a system-wide plan for rolling out the platform, which could include securing buy-in from administrative leadership, staff or patient training on how to use the platform, and identifying experts or advocates who could act as a go-to for all technology-related concerns (ie, access to mobile devices, difficulty logging in, and trouble with the interface) [22,25,26].

Finally, settings with a well-established EHR or patient portal that offers many of the same features, as OARS might not benefit from implementing an additional technology platform. Instead, platforms like OARS might be most appropriate in settings that want to implement a digital health tool for the first time to support their patients on MOUD or who experience limited patient engagement with treatment or existing platforms. In this way, OARS can offer an alternative means to reach patients and keep them engaged in care. However, the barriers noted in this paper (eg, lack of provider buy-in and EHR integration) might still jeopardize the implementation of digital health tools in diverse health care systems and will need to be addressed.

### Limitations

The findings should be interpreted within the context of our study limitations. First, this was a single-site case study where the digital health tool (OARS) was not integrated into the Center’s EHR. Future research is needed to understand the implementation facilitators and barriers within primary care settings, where the digital tool is integrated into the EHR to assess the utility of implementing a third-party technology platform to support the management of MOUD services. Second, this study was observational and lacked a control group, introducing selection bias into the sample of patients who used OARS. Third, the qualitative evaluation of this study involved a limited number of staff members. To understand whether the perspectives offered during the interviews were indicative of the overall staff experience implementing similar digital health tools in busy primary care settings, other approaches such as surveys or brief interviews could be conducted. Finally, contact between the study team and patients receiving MOUD at the Center was strictly prohibited in our case study. Therefore, our understanding of the value of OARS for patients is limited to the perceptions of providers who treat these patients. However, future projects seeking to implement a third-party technology platform like OARS may benefit from directly interviewing patients to understand their experiences using the system and how those experiences may align or contrast with the expectations of their providers.

### Conclusions

Our findings highlight many of the key challenges to be solved when integrating a third-party technology tool into existing EHR systems. Once proven efficacious, future research is needed to understand the potential utility of systems like OARS for improving outcomes (eg, adherence) for people with OUD.

## Abbreviations

EHR: electronic health record
MOUD: medication for opioid use disorder
OARS: Opioid Addiction Recovery Support
OUD: opioid use disorder
